# Effects of zeolite supplementation on parameters of intestinal barrier integrity, inflammation, redoxbiology and performance in aerobically trained subjects

**DOI:** 10.1186/s12970-015-0101-z

**Published:** 2015-10-20

**Authors:** Manfred Lamprecht, Simon Bogner, Kurt Steinbauer, Burkhard Schuetz, Joachim F. Greilberger, Bettina Leber, Bernhard Wagner, Erwin Zinser, Thomas Petek, Sandra Wallner-Liebmann, Tanja Oberwinkler, Norbert Bachl, Gert Schippinger

**Affiliations:** Institute of Physiological Chemistry, Medical University of Graz, Graz, Austria; Institute of Nutrient Research and Sport Nutrition, Graz, Austria; SportchirurgiePlus, Graz, Austria; Biovis Diagnostic MVZ GmbH, Limburg, Germany; Institute of Laboratory Sciences, Dr Greilberger GmbH, Laßnitzhöhe, Austria; Department of Surgery, Division of Transplantation Surgery, Medical University of Graz, Graz, Austria; FH JOANNEUM Ltd, Graz, Austria; Medical and Chemical Laboratory diagnostics Lorenz & Petek Ltd, Graz, Austria; Institute of Pathophysiology and Immunology, Medical University of Graz, Graz, Austria; Panaceo International Active Mineral Production GmbH, Villach, Austria; Institute of Sport Science, University of Vienna, Vienna, Austria

**Keywords:** Zeolite supplementation, Trained subjects, Leaky gut, Tight junctions, Zonulin

## Abstract

**Background:**

Zeolites are crystalline compounds with microporous structures of Si-tetrahedrons. In the gut, these silicates could act as adsorbents, ion-exchangers, catalysts, detergents or anti-diarrheic agents. This study evaluated whether zeolite supplementation affects biomarkers of intestinal wall permeability and parameters of oxidation and inflammation in aerobically trained individuals, and whether it could improve their performance.

**Methods:**

In a randomized, double-blinded, placebo controlled trial, 52 endurance trained men and women, similar in body fat, non-smokers, 20–50 years, received 1.85 g of zeolite per day for 12 weeks. Stool samples for determination of intestinal wall integrity biomarkers were collected. From blood, markers of redox biology, inflammation, and DNA damage were determined at the beginning and the end of the study. In addition, VO_2max_ and maximum performance were evaluated at baseline and after 12 weeks of treatment. For statistical analyses a 2-factor ANOVA was used.

**Results:**

At baseline both groups showed slightly increased stool zonulin concentrations above normal. After 12 weeks with zeolite zonulin was significantly (*p* < 0.05) decreased in the supplemented group. IL-10 increased tendentially (*p* < 0.1) in the zeolite group. There were no significant changes observed in the other measured parameters.

**Conclusions:**

Twelve weeks of zeolite supplementation exerted beneficial effects on intestinal wall integrity as indicated via decreased concentrations of the tight junction modulator zonulin. This was accompanied by mild anti-inflammatory effects in this cohort of aerobically trained subjects. Further research is needed to explore mechanistic explanations for the observations in this study.

## Background

Zeolite is a collective name for minerals and chemical compounds within the group of silicates. Natural zeolites are crystalline compounds of volcanic origin with microporous structures of Si- and AI-tetrahedrons (SiO_4_, AIO_4_), linked through the common oxygen atoms to form an open crystal structure [1]. The arrangement of the atoms affects the water-binding capacities of the microporous structures of the zeolite minerals, like a “rock-sponge”. The grid structure of zeolites can act as inorganic cation-exchanger, adsorbent, detergent builder, and active reservoir for metal-catalyzed reactions [2–6]. These properties have earned them extensive industrial applications. Zeolite’s use in medicine, especially micronized natural zeolite-clinoptilolite, is a relatively novel subject of interest. Animal studies have demonstrated that natural zeolite-clinoptilolite exerts immunostimulatory effects, modulates anti- and pro-inflammatory mechanisms, and postulate its use as an adjuvant in anticancer therapy [7, 8]. Other researchers describe zeolite’s capability to adsorb glucose, antidiarrheic effects, and its strong antioxidant activity [9–11].

Regarding sports nutrition, zeolite’s effects in the gut, its effects on inflammatory metabolism and antioxidant activity, and effects on performance are of specific interest. For instance, in performance sports there is a high prevalence of gastrointestinal complaints among endurance athletes like runners and triathletes [12]. These problems are attributed to changed blood flow that is shunted from the viscera to skeletal muscle or the heart [13]. Symptoms described are nausea, stomach and intestinal cramps, vomiting and diarrhea, and are accompanied by increased intestinal wall permeability. Such a “leaky gut” leads to endotoxemia, and results in increased susceptibility to infectious- and autoimmune diseases, due to absorption of pathogens/toxins into tissue and blood stream [14–16].

For this study, we hypothesized that the above described properties of zeolite are able to

- modulate intestinal wall integrity via impact on zonulin secretion from enterocytes,
- reduce pro-oxidative and pro-inflammatory processes in the gut via binding of oxidants,
- increase VO_2max_ and maximum performance as resulting outcomes of an improved gut wall integrity, redox balance and anti-inflammatory metabolism.

The effects of zeolites on intestinal barrier integrity, inflammation, redox biology, as well as on physical performance are not elucidated. From these perspectives, nutritional solutions like zeolite supplementation could be of reasonable relevance for athlete’s health and performance.

Hence, we focused the primary outcome of this study to explore the effects of a zeolite-clinoptilolite supplement on markers of intestinal barrier integrity in trained men and women. The secondary outcome of this trial was to evaluate whether the zeolite supplementation affects blood markers of redox biology and inflammation. A third hypothesis tested whether the zeolite supplementation could affect VO_2max_ and maximum performance in a step test ergometry.

## Methods

### Subjects

Fifty two endurance trained subjects, most of them cyclists and triathletes, participated in this trial. Inclusion criteria: healthy males or females, 25 – 50 years, aerobically trained (VO_2max_ from 45 mL/kg ^**.**^ min^-1^ to 65 mL/kg ^**.**^ min^-1^), non-smokers, no dietary or nutritional supplement use within four weeks prior to the first blood drawing/stool collection. Exclusion criteria: smokers, subjects outside the predefined VO_2max_ range, subjects who underwent major changes in training regimens during the study, chronic or excessive alcohol consumption, recent surgery or illness, body fat > 20 %. Body fat content and distribution was estimated by a computerized optical device Lipometer (Möller Messtechnik, Graz, Austria), as described by Möller, et al [17]. Besides inclusion and exclusion criteria, a standard blood chemistry panel was determined and all subjects completed a medical history.

### Ethical aspects, recruitment and randomization

All subjects provided written informed consent prior to participating in this investigation. This study was conducted according to the guidelines of the Declaration of Helsinki for Research on Human Subjects 1989 and was approved by the Ethical Review Committee of the Medical University of Graz, Austria. The trial was registered under www.clinicaltrials.gov, identifier: NCT01831492.

The study focused trained men and women and was announced in the largest sport magazine of Austria. After a telephone screening conducted by the research team, 61 subjects volunteered for eligibility testing. From those, 56 men and women were selected and entered the study program.

Before randomization VO_2max_ tests were conducted and VO_2max_ data were ranked from highest to lowest. Subjects were randomized into blocks of four and sequentially numbered. To guarantee a balanced VO_2max_ distribution between groups (zeolite versus placebo) we conducted stratification via VO_2max_ rank statistics. Randomization code was held by a third party (Union of Sport and Exercise Scientists Austria) and handed over for statistical analyses after collection of all data.

### Study design and time schedule

This was a randomized, placebo controlled, double-blinded study. All eligibility testing - such as blood panel, eligibility for exercise, clinic check-up, medical history questionnaire, one-on-one interview - was finalized prior to the 1^st^ VO_2max_ testing. On the day of the 1^st^ VO_2max_ testing blood and stool samples were collected before the subjects conducted the cycle step test ergometries (= first visit and 1^st^ VO_2max_ testing) to exhaustion. After completion of these 56 ergometer step tests, the investigators dispensed the randomized capsule supply according to the subject’s VO_2max_-ranking. After 12 weeks taking the capsules as instructed, subjects returned to the study side for the second blood and stool collection and another VO_2max_ testing, and returned their remaining capsules to adjust compliance.

### Dietary assessment

Subjects were instructed to maintain their habitual diet, lifestyle and training regimen during the 12 weeks study and to duplicate their diet before each blood/stool collection. Before the first visit at the study site, subjects completed a 7-day food record for nutrient intake assessment. They subsequently received copies of their 7-day diet records and were instructed to replicate the diet prior to the second visit. Diet records were analyzed for total calories, protein, carbohydrate, fat, cholesterol, fiber, water, alcohol, several vitamins, minerals, and fatty acids using “opti diet” software 5.0 (GOEmbH, Linden, Germany).

### Treatment

The subjects randomized to zeolite-clinoptilolite (n = 28) received boxes with capsules containing (per capsule): Zeolite 307,50 mg, Dolomite 75,17 mg (thereof Magnesium 15,79 mg; Calcium 34,9 mg), Maca 27,33 mg, Cellulose 90 mg (PANACEO SPORT®, Panaceo International Active Mineral Production GmbH, Villach, Austria). The placebo consisted of identical appearing capsules with 500 mg Cellulose per capsule. All subjects were instructed to take 6 capsules per day, 3 capsules in the morning with breakfast and the other 3 capsules with the last meal of the day throughout 12 weeks. To ensure good compliance, subjects were called and e-mailed biweekly to remind and motivate them to adhere to the suggested instructions.

### VO_2max_ and performance tests

To assess the supplement’s impact on VO_2max_ and maximum performance in watt (W), and to avoid biased responses caused by differences in aerobic fitness levels, we conducted step wise incremental ergometries until exhaustion to evaluate VO_2max_ and maximum performance of each subject, at the beginning and the end of the study. Exhaustion was reached/defined when cadence could not be maintained with maximum exertion on the last (heaviest) step. They were instructed not to perform physical training 3 days prior to the exercise test. All subjects performed an incremental cycle ergometer exercise test (EC 3000, Custo med GmbH, Ottobrunn, Germany) at 80 rpm. After a three minute rest phase sitting inactive on the ergometer, work rate started at 60 W (women 40 W) for three minutes and was increased 15 W every minute until voluntary exhaustion. A standard electrocardiogram was recorded during the entire test, which was supervised by a physician. Respiratory gas exchange variables were measured throughout the incremental exercise tests using a breath-by-breath mode (Metalyzer 3B, Cortex Biophysik GmbH, Leipzig, Germany). During these tests, subjects breathed through a facemask. Oxygen uptake (VO_2_), carbon dioxide output (VCO_2_), minute ventilation (V_E_), breathing rate (BR) and tidal volume (V_T_) were continuously obtained. Blood pressure as well as heart rate (HR) were monitored throughout the tests using a commercially available heart rate monitor (Polar Vantage NV, Polar Electro Finland).

### Feces and blood collection

For the measurements of zonulin and alpha1-antitrypsin from feces the subjects collected samples at baseline and after 12 weeks. They used standardized stool tubules and brought the samples with a cooling bag, within 24 h after collection, to the laboratory. All samples were analyzed within 48 h after dispensing. Throughout the 12 weeks treatment the subjects recorded a stool protocol to monitor stool appearance with help of the Bristol stool scale/chart [18].

Blood collections were conducted in supine position from a medial cubital vein at baseline and after 12 weeks of treatment. Venous blood was collected to determine carbonyl proteins (CP), 8-iso-prostaglandin F_2α_ (8-iso-PGF_2α_), plasma glutathione peroxidase (GPx3), uric acid, vitamin C, DNA strand-breaks, cytokines like tumor necrose factor alpha (TNF-α), interleukines 6, 8, 10, 22 (IL-6, IL-8, IL-10, IL-22). For determination of a blood clinical chemistry panel we measured Mg, Ca, Fe, K, Na, AI, P, Cl, ferritin, transferrin, liver and kidney parameters (ALT, AST, GGT, creatinine), testosterone, sex hormone binding globulin (SHBG), free androgen index (FAI), and a hemogram.

### Stool analyses

Zonulin and α1-antitrypsin were analyzed with commercially available ELISA kits (Immundiagnostik AG, Bensheim, Germany).

### Analyses of blood parameters

The quantity of carbonyls in protein samples (carbonyl proteins, CP) was analyzed with a commercially available ELISA kit from Immundiagnostik AG, Bensheim, Germany.

An isotope diluted liquid chromatography tandem mass spectrometry (LC-MS) approach was used for the detection of 8-iso-PGF_2α_.

For analyses of plasma GPx3, the Human Glutathione Peroxidase ELISA Kit assay was used (Cusabio, antibodies-online GmbH, Aachen, Germany).

For DNA strand-break analyses in mononuclear cells the Comet assay was used.

For analyses of cytokines IL-6, IL-8, IL-10 and IL-22, an ELISA kit was used (Cusabio; antibodies-online GmbH; Aachen, Germany).

For quantitative determination of TNF-alpha an enzyme-linked-immunosorbent Assay (TNF-alpha ELISA; Immundiagnostik AG, Bensheim, Germany) was used.

For analyses of vitamin C, uric acid, minerals, functional liver and kidney markers, as well as androgens, fully automated routine clinical chemistry analyzers were used (vitamin C: LaChrom L7200, Merck KG, Darmstadt, Germany; all the others: CI 8200, Abbott Diagnostics, Green Oaks, IL).

The hemogram was measured with the ABX Micros 60 (AxonLab, Baden, Switzerland) from EDTA-blood.

### Statistical analyses and sample size calculation

Per protocol analyses were performed using SPSS for Windows software, version 19.0. Data are presented as mean ± SD. Statistical significance was set at *P* < 0.05. The Shapiro-Wilk test was used to determine normal distribution. Baseline characteristics, nutrient and clinical chemistry data, were compared by unpaired Student’s *t*-test. Data obtained for zonulin and α1-antitrypsin from feces, as well as CP, 8-iso-PGF_2α_, GPx3, uric acid, vitamin C, DNA strand-breaks, IL-6, IL-8, IL-10, IL-22 from blood and performance data were analyzed using a univariate, two-factorial, repeated measures analysis of variance (ANOVA). Factors: treatment (zeolite supplementation versus placebo), time (baseline versus 12 weeks of treatment). Significant interactions and main effects were post-hoc analyzed by using Bonferroni correction. Performance data were analyzed separately for men and women.

Sample size calculation was based on zonulin, redox markers and cytokines. We estimated 12 (zonulin) - 25 (TNF-α) subjects per group (1 : 1 distribution) - depending on the calculated parameter, standard deviation and effect size - to reach a probability of error (alpha/2) of 5 % (α = 0.05) and 80 % power. TNF-α sample size calculation resulted in the highest number of all parameters: 50 subjects. Hence, and including a drop-out rate of 10 %, 56 subjects were recruited.

## Results

### Study population, baseline characteristics, nutrition

**Fig. 1:**
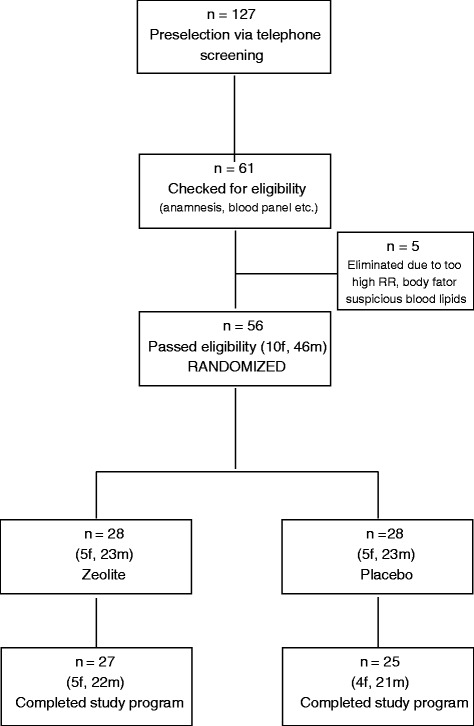
CONSORT diagram

A CONSORT diagram outlining participant recruitment is depicted Fig. 1. Of the 56 randomized subjects, 52 completed the full program and entered statistical analyses (9 women, 43 men). Two men terminated earlier due to injuries unrelated to the study, one men became sick with influenza, and one woman did not adhere to the strict exclusion of any supplement use.

Returned capsule count after the treatment period revealed a compliance >90 % in both groups. Groups did not differ in age, gender, BMI, VO_2max_, body weight and fat, clinical blood chemistry variables, and diet (*P* > 0.05). Subjects characteristics are presented in Table 1.

**Table 1:** Group characteristics, performance data, clinical chemistry and nutrition data of 52 trained subjects^a^, estimated at baseline

Variable	Reference range^b,c^	Zeolite	Placebo
		(n = 27)	(n = 25)
Group characteristics, VO_2max_, P_max_ data:			
Age, yr		33.6 ± 5.3	37.2 ± 4.9
BMI, kg ^.^ m^-2^		22.7 ± 2.2	23.2 ± 2.1
Weight, kg (men)		73.8 ± 5.9	74.6 ± 6.3
Weight, kg (women)		56.7 ± 3.9	58.8 ± 3.4
Total body fat, % (men)		13.5 ± 3.1	14.1 ± 3.9
Total body fat, % (women)		17.7 ± 2.1	17.3 ± 2.2
VO_2max_, mL (men)^d^		4263 ± 132	4187 ± 119
relVO_2max_, mL ^.^ kg^-1 .^ min^-1^(men)^e^		53.2 ± 5.1	52.3 ± 4.6
VO_2max_, mL (female)^f^		2367 ± 128	2357 ± 132
relVO_2max_, mL ^.^ kg^-1 .^ min^-1^(women)^g^		42.5 ± 3.6	41.4 ± 3.6
P_max_, W (men)^h^		360.4 ± 29.1	352.3 ± 24.6
relP_max_, W ^.^ min^-1^ (men)^i^		4.6 ± 0.5	4.5 ± 0.4
P_max_, W (women)^j^		240.5 ± 25.5	241.8 ± 23.6
relP_max_, W ^.^ min^-1^ (women)^k^		4.2 ± 0.8	4.1 ± 0.7
Clinical Chemistry (exerpts):			
Glucose, mmol ^.^ L^-1^	3.9 - 6.1	4.1 ± 0.5	4.6 ± 0.4
Hemoglobin, g ^.^ L^-1^	136 - 172	152 ± 12	150 ± 19
Iron, μmol ^.^ L^-1^	11 - 32	21.4 ± 5.5	20.6 ± 4.9
Ferritin, μg ^.^ L^-1^	18 - 300	111 ± 32	99 ± 26
Cholesterol, mmol ^.^ L^-1^	<5.85	4.27 ± 1.03	4.36 ± 1.13
HDL, mmol ^.^ L^-1^	0.80 - 1.80	1.20 ± 0.13	1.23 ± 0.19
Triglycerides, mmol ^.^ L^-1^	<1.80	0.92 ± 0.32	0.98 ± 0.26
Testosterone, nmol ^.^ L^-1^(men)	10 - 31	16.3 ± 4.9	18.2 ± 4.1
Creatinine, μmol ^.^ L^-1^	50 - 110	90 ± 11	89 ± 17
Vitamin C (ascorbate), μmol ^.^ L^-1^	30 - 110	57.3 ± 15.7	63.6 ± 22.9
Uric acid, μmol ^.^ L^-1^	120 - 420	315.5 ± 69.6	311.4 ± 64.4
Aluminium, nmol ^.^ L^-1^	0 - 560	199.2 ± 43.6	209.1 ± 59.3
Nutrition (exerpts):			
Energy, kJ ^.^ d^-1^	11776 - 13902	12445 ± 893	12756 ± 1455
Fat, %	<30 % of kJ · d^-1^	33.5 ± 4.2 %	34.1 ± 3.1 %
Protein, %	10 - 15 % of kJ · d^-1^	16.3 ± 2.1 %	15.8 ± 3.2 %
Carbohydrates, %	>50 % of kJ · d^-1^	48.3 ± 9.1 %	49.2 ± 10.3 %
Water, mL	2600	2998 ± 495	2779 ± 682
Fibres, g	30	26 ± 5	25 ± 4
Vitamin C, mg	72 - 106	132 ± 48	118 ± 67
Vitamin E, mg	14	15 ± 5	13 ± 9
Folate, μg	434 - 505	282 ± 155	292 ± 165
Vitamin B-6, mg	3.2 - 3.8	1.89 ± 2.9	1.85 ± 2.8
Vitamin B-12, μg	3.3 – 3.7	5.0 ± 2.8	5.6 ± 1.4
Sodium, mg	>646	2711 ± 885	2689 ± 970
Potassium, mg	2171 - 2523	3362 ± 904	3436 ± 1251
Magnesium, mg	185 - 361	408 ± 134	385 ± 119
Calcium, mg	1085 - 1261	1080 ± 395	1007 ± 327
Iron, mg	10.9 - 12.5	14.3 ± 5.6	13.9 ± 7.2

^a^Values are means ± SD, and did not differ between the groups (*P* > 0.05, Student’s *t*-test); ^b^Reference range for clinical chemistry parameters [31]; ^c^Reference values for dietary intake (RDA) in Germany, Austria, Switzerland [32], ranges presented here apply to physical active people; *VO*_*2max*_ maximum oxygen uptake, *P*_*max*_ maximum performance; ^d-k^No effects of 12 weeks of treatment or time on VO_2max_, relVO_2max_, P_max_, or relP_max_, neither in men nor in women

### Markers of intestinal barrier permeability

**Fig. 2:**
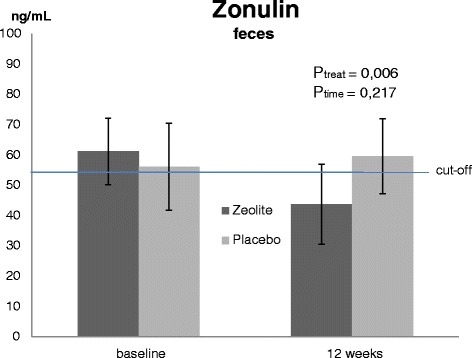
Stool concentrations of zonulin in trained subjects before and after 12 weeks of treatment. Values are means ± SD, *P* < 0.05 (ANOVA), n = 27 (zeolite supplementation), n = 25 (Placebo)

Zonulin was determined from feces from the last stool prior to the visit at the study site. There were no significant differences between groups at baseline (*P* > 0.1) but the mean values of both groups were slightly above cut-off (ref. range: < 55 ng ^**.**^ mL^-1^, see Fig. 2). After 12 weeks with zeolite zonulin decreased in the verum group into normal physiological range (43.8 ± 14.3 ng ^**.**^ mL^-1^) whereas the placebo group did not really change over time (59.6 ± 12.3 ng ^**.**^ mL^-1^). This revealed a significant difference between groups/treatments (*P* = 0.006) and is corresponding to a decrease of almost 30 % in the zeolite supplemented group (Table 2).

**Table 2:** Summary of results*; gut barrier integrity markers, oxidation markers, inflammatory markers

Variable	P_treat_	P_time_	Zeolite	Placebo	Zeolite	Placebo	Reference range
			0 week	0 week	12 weeks	12 weeks	
zonulin	0.006	>0.1	61.2 ± 11.0	56.1 ± 13.2	43.8 ± 14.3	59.6 ± 12.3	<55
ng ^.^ mL^-1^							
α1-antitrypsin	>0.1	>0.1	27.6 ± 14.8	32.8 ± 15.6	30.2 ± 18.1	35.8 ± 17.7	<37.5
mg ^.^ dL^-1^							
CP	>0.1	<0.001	218 ± 102	206 ± 98	508 ± 108	486 ± 87	80–200
pmol ^.^ mg^-1^							
iPF2α-III	>0.1	>0.1	216 ± 55	200 ± 55	199 ± 73	181 ± 43	50–400
pg ^.^ mL^-1^							
GPx3	>0.1	<0.001	131 ± 69	107 ± 58	182 ± 64	193 ± 59	33–98
IU ^.^ L^-1^							
DNA SB	>0.1	>0.1	0.36 ± 0.25	0.35 ± 0.24	0.49 ± 0.33	0.48 ± 0.29	0–70
AU							
TNF-α	>0.1	0.024	26.1 ± 8.7	24.3 ± 12.3	31.8 ± 11.5	28.7 ± 11.8	0–69
pg ^.^ mL^-1^							
IL-6	>0.1	>0.1	26.6 ± 2.0	27.1 ± 1.7	27.7 ± 3.2	26.9 ± 2.7	<100
pg ^.^ mL^-1^							
IL-8	>0.1	>0.1	38.1 ± 10.8	41.9 ± 12.0	39.7 ± 11.5	39.8 ± 9.7	0–62
pg ^.^ mL^-1^							
IL-10	0.087	>0.1	40.1 ± 19.2	42.1 ± 16.0	54.5 ± 27.8	37.9 ± 22.2	none
pg ^.^ mL^-1^							
IL-22	>0.1	>0.1	42.9 ± 4.2	41.4 ± 3.3	40.5 ± 3.3	40.1 ± 4.4	none
pg ^.^ mL^-1^							

*Values are means ± SD; *P* < 0.05, ANOVA; n = 27 (Zeolite), n = 25 (Placebo); *α1-antitrypsin* alpha1-antitrypsin, *CP* carbonyl groups bounded on protein, *iPF2α-III* isoprostanes F2α-III, *GPx3* plasma glutathione peroxidase, *DNA SB* DNA strand-breaks, *TNF-α* tumor necrose factor alpha, *IL* interleukin, *treat* treatment

Alpha1-antitrypsin: There were no differences between groups at any time point assessed (*P* > 0.1). Mean concentrations in feces were within normal range at baseline and after 12 weeks of treatment (<37.5 mg ^**.**^ dL^-1^, Table 2).

### Redox markers

CP: There were no differences in CP concentrations between groups, neither at baseline nor after 12 weeks of treatment (*P* > 0.1). There was a significant effect of time (*P* < 0.001) to increased values in both groups at the end of the study. Whereas CP mean concentrations were only slightly above cut-off (200 pmol ^**.**^ mg^-1^) at baseline, the concentrations were around 500 pmol ^**.**^ mg^-1^ after 12 weeks (Table 2).

8-iso-PGF_2α_ did not show differences between groups at any time point assessed (*P* > 0.1). 8-iso-PGF_2α_ mean concentrations were within normal range at baseline and after 12 weeks of treatment (50 - 400 pg ^**.**^ mL^-1^, Table 2).

Plasma GPx3 activities did not differ between groups at any time point assessed (*P* > 0.1). There was a significant effect of time (*P* < 0.001) to increased values at the end of the study. Whereas GPx3 mean values were only slightly above cut-off (98 IU ^**.**^ L^-1^) at baseline, the concentrations were close to 200 IU ^**.**^ L^-1^ after 12 weeks (Table 2).

DNA strand-breaks did not show differences between groups at any time point assessed (*P* > 0.1). All values were within normal range at baseline and after 12 weeks of treatment (0 – 70 AU, Table 2).

Vitamin C and uric acid did not show differences between groups, neither at baseline (Table 1) nor after 12 weeks of treatment (*P* > 0.1, data not shown). All values were within normal ranges at both time points assessed.

### Markers of inflammation

TNF-alpha concentrations did not differ between groups at both time points assessed (*P* > 0.1). There was a significant effect of time (*P* = 0.024) to increased values in both groups at the end of the study. All values were within normal range at baseline and after 12 weeks of treatment (0 – 69 pg ^**.**^ mL^-1^, Table 2).

The pro-inflammatory cytokines IL-6, IL-8 and IL-22 did not show differences between groups at any time point assessed (*P* > 0.1). All these cytokines concentrations were either within normal ranges at baseline and after 12 weeks of treatment or unobtrusive (Table 2).

Anti-inflammatory IL-10: There were no significant differences between groups at baseline (*P* > 0.1). After 12 weeks with zeolite IL-10 increased in the zeolite group whereas the placebo group did not really change over time. This revealed a tendential difference between groups/treatments (*P* = 0.087, Table 2) at the end of the study.

### Performance data

VO_2max_ and P_max_ did not differ between treatment groups at any time point assessed (*P* > 0.1), neither in men nor in women. Also the factor time (baseline – 12 weeks) had no influence on these parameters (*P* > 0.1).

### Other blood analytes

Blood minerals (including aluminum), liver and kidney parameters, androgen status, and hemogram did not show differences between groups, neither at baseline (excerpts see Table 1) nor after 12 weeks of treatment (*P* > 0.1, data not shown). All values were within normal ranges at both time points assessed.

## Discussion

This study evaluated whether zeolite supplementation could influence biomarkers of gut wall and tight junction integrity, oxidation and inflammation in aerobically trained individuals.

### Markers of intestinal barrier permeability

Tight junctions are protein structures that represent the major barrier within the intestinal paracellular pathway and are therefore key factors of intestinal permeability. They regulate the movement of fluid, macromolecules and leukocytes between the bloodstream and the intestinal lumen, and vice versa [19].

Zonulin - a protein of the haptoglobin family - is described as the main physiological modulator of intercellular tight junctions. Increased zonulin concentrations are related to changes in tight junction competency and increased intestinal permeability [20]. The “leak” in the paracellular absorption route enables antigens to pass from the intestinal milieu, challenging the immune system to produce an immune response and subsequent inflammation and oxidative stress [19, 21, 22]. Beside liver cells, intestinal cells can synthesize zonulin and the zonulin system can be activated by dietary proteins (especially gliadin) or enteric bacteria [20, 23].

Alpha1-antitrpysin is an acute phase protein synthesized in liver cells. Its concentrations in feces serve as marker for intestinal barrier integrity and is widely used to estimate protein leakage into the intestinal tract [24, 25]. In this study we observed significant reduced stool zonulin concentrations after zeolite treatment but alpha1-antitrypsin did not change after treatment and was within reference range throughout the study. We are convinced that, although our subjects showed indices of a mild gut permeability disturbance at baseline (zonulin slightly above cut-off), this mild imbalance in gut barrier function was not distinctive enough to provoke an acute-phase response in liver cells via increased alpha1-antitrypsin synthesis.

Nevertheless, the observed reduction of zonulin in the zeolite supplemented group is remarkable as mean concentrations dropped from above cut-off into reference range (<55 ng ^**.**^ mL^-1^). These data indicate that our trained cohort suffered already a mild increase in intestinal permeability at baseline, probably due to chronic exercise training. Obviously the 12 weeks of zeolite supplementation could reduce zonulin synthesis and hence improve intestinal barrier integrity. Therefore we suggest that the zeolite modulated the zonulin system, perhaps by interacting with intestinal bacteria.

However, we have to admit that the mechanisms how zeolite affects intestinal barrier are unclear. We believe that one or more of the described zeolite properties exerted in the intestinal tract like immunostimulation, impact on inflammation, adsorbent or detergent properties, might have contributed to this result.

### Redox markers

CP and GPx3 showed a significant influence of time with higher values in both groups at the end of the study. Basically, higher concentrations in CP in trained cohorts and in relation to exercise are not new for us and we observed and discussed this recently [26, 27]. But in this study subjects were instructed to avoid changes in their training regimens and lifestyles throughout the treatment period and thus we cannot relate the increase of these markers to a change in subject’s exercise training regimens or stress profile. We suppose rather a mild nutritional influence. The baseline values were collected in fall and the study end was between January and March. Although subjects were asked to avoid changes in food intake, seasonal changes could lead to decreased micronutrient and antioxidant intake over winter time which in turn could have led to decreased protection of proteins from oxidation and internal up-regulation of GPx3 activity to compensate redox state in plasma. Insofar this is a realistic hypothesis as we know that a treatment with higher concentrations of isolated antioxidants can decrease plasma GPx3 activities within 3 weeks [28]. On the contrary, interview data revealed that the subjects adhered to the instruction to replicate their nutrition the last days before visiting the study site for the second time. Also vitamin C and uric acid, the main determinants of plasma redox status, were unchanged after 12 weeks. However, this does not exclude dietary changes in the intake of other, especially redox-active, nutrients over the winter months.

Regarding the question whether a 1.85 g zeolite supplementation could affect redox markers in healthy trained subjects, we have to falsify this hypothesis. None of the measured redox markers CP, GPx, 8-iso-PGF_2α_, vitamin C and uric acid did react to the applied zeolite treatment. Also DNA strand-breaks in peripheral mononuclear cells did not show any changes. Obviously the mild zeolite modulations in the intestinal wall did not affect systemic redox markers in the blood.

### Markers of inflammation

TNF-alpha, a marker of low-grade inflammation, was not different between groups but increased towards the end of the study in both groups. This is similar to CP and GPx3 and we already observed a correlation between CP and TNF-alpha in the past [29], but related this to common cold pathophysiology. We do not have an explanation for this increase other than this change might also be related to a reduced intake of food high in micronutrients and antioxidants over winter time. Nevertheless, although TNF-alpha concentrations increased, all values observed were within the reference range and thus, we think that the changes might be due to biological variability.

The lack of inflammatory response to the treatment is in line with the unobtrusive concentrations of the inflammatory cytokines IL-6, IL-8, and IL-22, with no differences between groups. In contrast the anti-inflammatory IL-10 showed an increase by a trend in the zeolite supplemented group compared to placebo. This could reflect an anti-inflammatory modulation which could be linked to the reduced intestinal wall permeability in the zeolite group as indicated by significant zonulin decrease after 12 weeks.

### Performance data

In recent years more and more athletes from the field reported to us about ergogenic effects after intake of zeolite. Despite the lack of a comprehensible biological rationale for this serious obeservations we decided to evaluate the effects of zeolite supplementation on VO_2max_ and/or P_max_ via exhaustive step wise incremental ergometry. With this double-blinded, placebo-controlled study design and the model of standardized performance testing applied in this trial, we could not detect differences in VO_2max_ and/or P_max_ after 12 weeks of supplementation. However, based on our results we cannot generally exclude ergogenic effects of zeolite. Perhaps another model of performance testing or the same experiment conducted with another cohort, e.g. with sedentary subjects, could reveal different results. Further, the application of a higher dose than used in our trial should be investigated. Our subjects received ~ 1.85 g zeolite per day (6 capsules), which corresponds to the lowest range of the vendor’s daily recommended dose: it ranges from 1.85 g to 2.7 g per day, corresponding to 6 – 9 capsules for healthy and recreationally active individuals in order to promote health and performance. Thus, additional research is needed to ascertain under which conditions zeolite might be ergogenic or not.

### Other blood parameters

As zeolites also contain aluminum [8, 11] we measured serum AI, liver and kidney parameters at the beginning and the end of the study to identify possible toxic effects. We did not observe any changes in AI, AST, ALT, GGT, or creatinine concentrations. The zeolite-clinoptilolite supplement we used in this study had a low AI content, according to the available (cyto-)toxicity certificates this was 11 %, which does obviously not affect serum AI concentrations or key hepatic markers when ingested over 3 months. This also indicates that the AI absorption from this product was not of pathophysiological relevance.

### Limitations of the study

First, we did not conduct food recording towards the end of the study to assess nutrient intake for a second time. Another dietary analysis at the end of the treatment period could have revealed some evidence whether our hypothesis of reduced micronutrient and antioxidant intake over winter time could have substance or not.

Second, the number of valid biomarkers we used to estimate intestinal barrier integrity was limited. For future studies, also other biomarkers such as lipopolysaccharides or the lactulose-mannitol/rhamnose intestinal permeability test could be used.

Third, the determination of an enteric bacteria profile and a microbiome analysis would be useful to correlate such analyses to changes in our outcome measures.

## Conclusions

Zeolite supplementation can beneficially affect intestinal barrier integrity, accompanied by mild anti-inflammatory effects in subjects undergoing regular aerobic exercise training. Redox markers in blood, VO_2max_ and P_max_ of trained subjects are not affected by ~1.85 g zeolite supplementation over 3 months and the applied model of performance testing. However, our observations could be of practical relevance for athletes under the perspective that an improved intestinal barrier reduces athlete’s susceptibility to endotoxemia [30]. Further research is needed to explore mechanistic explanations for the observations in this study.

## Abbreviations

AI: Aluminum
ALT: Alanine-aminotransferase
AST: Aspartate-aminotransferase
ANOVA: Analysis of variance
AU: Arbitrary units
BMI: Body mass index
BR: Breathing rate
CONSORT: Consolidated standards of reporting trials
CP: Carbonyl groups bounded on protein
DNA: Deoxyribonucleic acid
DNPH: Dinitrophenylhydrazine
EDTA: Ethylenediaminetetraacetic acid
ELISA: Enzyme linked immunosorbent assay
FAI: Free androgen index
GPT: Glutamate-pyruvate-transaminase
GPx3: Plasma glutathione peroxidase
HR: Heart rate
IL: Interleukin
iPF2α-III: Isoprostanes F2α-III
IU: International unit
PBS: Phosphate buffer saline
Rpm: Randoms per minute
SD: Standard deviation
SHBG: Sex hormone binding globulin
Si: Silicium
TNF-alpha: Tumor necrosis factor alpha
V_E_: Ventilation per minute
VCO_2_: Carbon dioxide output
VO_2max_: Maximum oxygen uptake
V_T_: Tidal volume
W: Watt
